# Cell Adhesion Molecules and Protein Synthesis Regulation in Neurons

**DOI:** 10.3389/fnmol.2020.592126

**Published:** 2020-11-12

**Authors:** Irina Kozlova, Saroj Sah, Ryan Keable, Iryna Leshchyns’ka, Michael Janitz, Vladimir Sytnyk

**Affiliations:** School of Biotechnology and Biomolecular Sciences, The University of New South Wales, Sydney, NSW, Australia

**Keywords:** cell adhesion molecules, neurons, transcription factors, gene expression, translation, endoplasmic reticulum, golgi apparatus

## Abstract

Cell adhesion molecules (CAMs) mediate interactions of neurons with the extracellular environment by forming adhesive bonds with CAMs on adjacent membranes or *via* binding to proteins of the extracellular matrix. Binding of CAMs to their extracellular ligands results in the activation of intracellular signaling cascades, leading to changes in neuronal structure and the molecular composition and function of neuronal contacts. Ultimately, many of these changes depend on the synthesis of new proteins. In this review, we summarize the evidence showing that CAMs regulate protein synthesis by modulating the activity of transcription factors, gene expression, protein translation, and the structure and distribution of organelles involved in protein synthesis and transport.

## Introduction

Protein synthesis in eukaryotes begins with the RNA polymerase II-mediated transcription of protein-coding genes in the nucleus of the cell. The RNA transcripts then undergo post-transcriptional modifications, that include splicing, capping, and polyadenylation (Ben-Yishay and Shav-Tal, 2019). The mature messenger RNA (mRNA) molecules are then exported from the nucleus to the cytosol *via* the nuclear pore complex (Xie and Ren, 2019). In the cytoplasm, mRNA is either degraded or stabilized, localized and used as a template for protein translation on ribosomes (Martin and Ephrussi, 2009; Keene, 2010). The endoplasmic reticulum (ER) is the main site of translation of cytosolic and membrane proteins by ribosomes located on its surface, although some of the cytosolic proteins are also translated on cytosolic ribosomes (Reid and Nicchitta, 2015).

The highly polarized morphology and function of neurons demand the modification of proteomes locally in axons and dendrites (Glock et al., 2017; Rangaraju et al., 2017). Ribosomes and ER accumulate in somata of neurons but are also distributed along dendrites and axons and are present at synapses. This spatial distribution enables protein synthesis not only in somata but also in dendrites and axons (Steward and Levy, 1982; Holt and Schuman, 2013). The local protein translation in dendrites is particularly active near synapses (Aakalu et al., 2001). It is initiated in response to stimuli inducing synaptogenesis and is required for synaptic plasticity. An extensive overview of the literature on the local protein synthesis in neurons can be found in several recent reviews (Rangaraju et al., 2017; Holt et al., 2019; Pushpalatha and Besse, 2019).

Integral membrane proteins made in the ER concentrate at specialized ER exit sites (ERESs), which are present in the ER throughout the somatodendritic compartment. Proteins are then delivered to the Golgi apparatus (Horton and Ehlers, 2003). The neuronal Golgi apparatus primarily localizes in the neuronal somata although Golgi cisternae also extend into dendrites. Discrete dendritic Golgi structures termed Golgi outposts are found in distal dendrites particularly at branch points but are excluded from axons (Horton et al., 2005; Ye et al., 2007). In the soma, proteins synthesized in the ER are delivered to the Golgi apparatus, sorted, and delivered to other neuronal compartments in the Golgi-derived vesicles. In contrast, proteins synthesized locally in the dendritic ER can be sorted *via* Golgi outposts (reviewed in Ramirez and Couve, 2011; Ehlers, 2013; Valenzuela and Perez, 2015). The Golgi-independent trafficking of locally synthesized proteins *via* recycling endosomes in dendrites has also been described (Bowen et al., 2017).

Neuronal growth, synapse formation, and function are regulated by cell adhesion molecules (CAMs). These cell surface glycoproteins have large extracellular domains, which mediate the interactions between neurons and the extracellular environment by forming adhesive bonds with proteins located on neighboring cells or in the extracellular matrix. Neurons express multiple families of CAMs (Shapiro et al., 2007). Members of the immunoglobulin superfamily (IgSF) and cadherins are characterized by the presence of the immunoglobulin-like and cadherin domains, respectively, and can form either homophilic adhesive bonds by binding to CAMs of the same type or heterophilic adhesive bonds by interacting with CAMs of a different type. Heterophilic adhesive bonds are also formed by other CAMs, such as post-synaptic neuroligins and presynaptic neurexins, or integrins, which bind to proteins of the extracellular matrix. CAMs are the carriers and receptors for glycans, which modulate the formation of adhesive bonds and interactions of CAMs with other extracellular proteins (Sytnyk et al., 2020). By forming adhesive bonds, CAMs mechanically stabilize synapses. They also assemble the transsynaptic scaffold recruiting other scaffolding proteins, neurotransmitter receptors, and different components of the synaptic machinery. Thereby, CAMs modulate the formation, maturation, stability, and strength of synapses (reviewed in Martin and Kandel, 1996; Togashi et al., 2009; Sytnyk et al., 2017; Tan et al., 2017; Keable et al., 2020). Also, CAMs initiate multiple intracellular signaling cascades in response to binding to their extracellular ligands (Juliano, 2002; Maness and Schachner, 2007; Leshchyns’ka and Sytnyk, 2016a). In the following sections, we summarize the current evidence indicating that the CAM-mediated signaling modulates the protein synthesis machinery, which produces proteins required for CAM-dependent changes in neuronal growth and function.

## CAMs Regulate Transcription

The idea that CAMs regulate gene expression was suggested by studies showing that the formation of cell-to-cell or cell-to-extracellular matrix contacts results in changes in gene expression. Dissociation of retina tissues results in a rapid decline in cortisol-induced mRNA expression of glutamine synthetase. This effect is reversed when cells are allowed to re-establish contacts with other cells (Vardimon et al., 1988). Disruption of cell-to-cell contacts in the *Xenopus laevis* embryo causes a decrease in α-actin mRNA levels (Sargent et al., 1986). When cultured on plastic, primary mouse mammary epithelial cells do not synthesize milk proteins but regain this ability when cultured on the Engelbreth-Holm-Swarm tumor matrix, laminin or heparan sulfate proteoglycans (Li et al., 1987).

Aggregation of chicken embryo brain cells causes changes in the transcription of several genes, including genes coding for the IgSF CAMs the neural cell adhesion molecule (NCAM), and neuron-glia cell adhesion molecule (Ng-CAM). This effect is inhibited by preventing cell aggregation with anti-NCAM Fab’ fragments (Mauro et al., 1994), indicating that changes in gene expression are induced by this CAM. Other CAMs also regulate transcription. The loss of L1, an IgSF CAM, in the brains of L1-deficient mice causes a reduction in the mRNA levels of microtubule-associated protein 2 (MAP2; Poplawski et al., 2012). Microarray analysis in the hippocampus of mice with ablated expression of neuronal growth regulator 1 (NEGR1), another IgSF CAM, identified 21 upregulated and 54 downregulated genes (Noh et al., 2019). Transcriptome sequencing identified 310 and 119 genes differentially expressed in the hippocampus of 22- and 66-day-old mice deficient in cadherin 13, respectively, indicating that the CAM-dependent regulation of transcription is developmentally regulated (Kiser et al., 2019).

Transcriptional changes observed in the brains of transgenic mice can also reflect the overall anatomical and functional changes in the brain. For example, the information processing mediated by immediate-early gene expression is altered in NCAM-deficient mice. In these animals, novel taste causes increased mRNA expression of a DNA-binding regulator protein c-fos in the amygdala, neutral taste causes increased mRNA expression of the activity-regulated cytoskeleton-associated protein (Arc) in the dentate gyrus, whereas the novelty-induced Arc increase in the cingulate cortex is inhibited (Montag-Sallaz et al., 2003). However, direct activation of CAMs *via* induction of homophilic adhesion or by using artificial ligands also results in changes in gene expression. In cultured astrocytes, the application of soluble NCAM purified from the early postnatal rat brain, which homophilically binds to the cell surface NCAM, induces changes in expression of 75 genes including an increase in mRNA levels of glutamine synthetase and calreticulin (Crossin et al., 1997). In rat hippocampal precursor cells, soluble NCAM induces an increase in the transcript levels of NR1 and GluR1, subunits of NMDA and AMPA receptors, respectively (Shin et al., 2002). Antibodies against the extracellular domain of NCAM used as an artificial ligand trigger the expression of Nr2f6, Lrp2, and Snca in cultured cerebellar neurons (Westphal et al., 2017b). Altogether, these studies indicate that CAMs are directly involved in the regulation of transcription.

## CAMs Regulate Transcription Factors

In cultured astrocytes, activation of NCAM with soluble NCAM induces an increase in the activity of promoters containing glucocorticoid response elements (Crossin et al., 1997). In cultured rat cerebellar neurons and rat forebrain astrocytes, purified NCAM, the IgIII domain of NCAM or antibodies against NCAM induce increased binding of the NF-kB family of transcription factors to DNA and increased transcription of the NF-kB responsive genes, such as *IkB-α* (Krushel et al., 1999; Table 1). In rat hippocampal precursor cells, soluble NCAM activates expression of transcription factors Neurogenin 1 (Ngn1) and NeuroD but decreases expression of Hes5 (Shin et al., 2002). The formation of neurites induced by homophilic interactions of NCAM in PC12-E2 cells is inhibited by overexpression of HES-1, a transcription repressor (Jessen et al., 2003). Altogether, these data indicate that NCAM regulates transcription by changing the expression and activities of transcription factors and transcription repressors in a cell type-specific manner. Other CAMs also regulate transcription factors (Table 1). Levels of inducible transcription factors, including neuronal PAS domain protein 4 (NPAS4), are reduced in embryonic cortical neurons derived from mice with ablated expression of a CAM amyloid precursor protein (Opsomer et al., 2020). In mouse cortical organoids deficient in contactin-associated protein-like 2 (Cntnap2), a member of the neurexin CAM family, levels of Dlx2, Nkx2.1, Ascl1, NeuroD, and Neurog2 transcription factors are reduced (Hali et al., 2020). In *Drosophila*, knock-down of the CAM klingon (Klg) causes a decrease in levels of a glial-specific paired-like homeodomain transcription factor Repo (Matsuno et al., 2015).

**Table 1:** Examples of transcriptional regulation by cell adhesion molecules (CAMs).

CAM	Transcriptional regulator/Mode of regulation	Cell type	Examples of the regulated gene(s)	Functional outcome	Reference(s)
APP	Not known	Mouse embryonic cortical neurons	Promotes the expression of NPAS4, downregulates GAD65, increases GABARα1	A decrease in the production of inhibitory neurotransmitter GABA	Opsomer et al. (2020)
Cntnap2	Not known	Mouse cortical organoids	Promotes the expression of Dlx2, Nkx2.1, Ascl1	GABAergic neurons production	Hali et al. (2020)
		Mouse cortical organoids	Promotes the expression of NeuroD, Neurog2	Neuronal differentiation and migration	
Klg	Not known	*Drosophila* glial cells	Promotes the expression of repo	Long-term memory formation	Matsuno et al. (2015)
L1	Activates MAPK	Mouse hippocampal neurons	Increases MAP2	Neurite outgrowth	Poplawski et al. (2012)
NCAM	Increases binding of NF-kB to DNA	Rat cerebella neurons and in neonatal forebrain astrocytes	Increases IkB-a	Not known	Krushel et al. (1999)
	Not known	Rat hippocampal precursor cells	Promotes the expression of Ngn1, NeuroD, NR1, and GluR1, inhibits expression of Hes5	Differentiation to glutamatergic neuronal cell type	Shin et al. (2002)
	Activates MAPK pathway, phosphorylates CREB	Rat hippocampal precursor cells, cultured dopaminergic, hippocampal, cerebral granule neurons, in PC12-E2 and rat neuroblastoma cell lines	Not known	Neurite outgrowth	Schmid et al. (1999); Kolkova et al. (2000); Shin et al. (2002); Neiiendam et al. (2004); Aonurm-Helm et al. (2008); Ditlevsen et al. (2008)
Negr1	Activates MAPK pathway	Mouse embryonic cortical neurons	Not known	Neurite outgrowth	Pischedda and Piccoli (2015)

**Figure 1:**
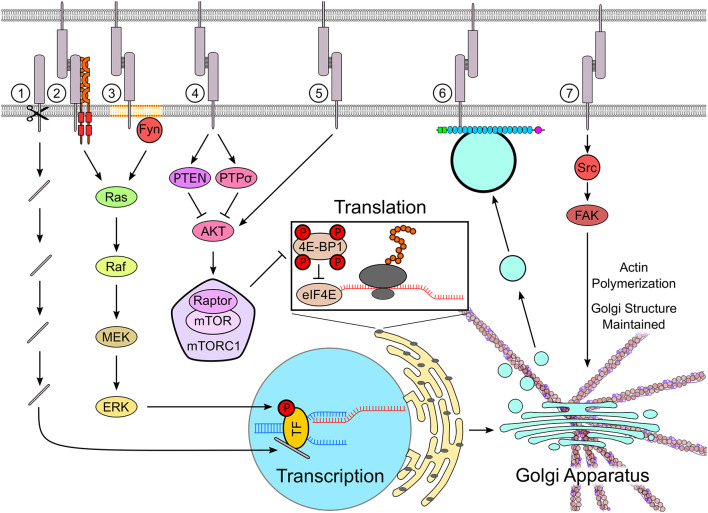
Cell adhesion molecules (CAMs) regulate protein synthesis machinery. CAMs such as neural cell adhesion molecule (NCAM), down syndrome cell adhesion molecule (DSCAM), DSCAM-Like-1 (DSCAML1), and L1 are cleaved by proteases, releasing fragments containing their intracellular domains, which are transported into the nucleus (1). In the nucleus, the CAM-derived fragments regulate the transcription of genes involved in neuronal differentiation and synapse formation by binding to transcription factors. Several CAMs, including NCAM, L1, and N-cadherin, activate the MAPK pathway, which results in the phosphorylation of transcription factors such as cyclic AMP response-element binding protein (CREB), thereby regulating transcription (2). NCAM activates the mitogen-activated protein kinase (MAPK) pathway by clustering and activating fibroblast growth factor receptor (FGFR) at the cell surface (2) and by activating an Src family kinase Fyn in lipid rafts (3). The mammalian target of rapamycin (mTOR) pathway, which controls the rate of translation, can be either activated or inhibited by CAMs. Homophilic binding of NB3 inhibits the mTOR pathway *via* PTPσ, whereas neuroligin-3 inhibits the mTOR pathway in cultured neurons and decreases the rate of protein translation by stabilizing the mTOR repressor PTEN (4). Adhesion molecule on glia (AMOG) increases cell size, when expressed in human glioma cells, and activates mTOR in these cells by promoting Akt phosphorylation independently of PI3K (5). The trafficking of newly synthesized proteins *via* TGN-derived organelles is regulated by NCAM, which binds to these organelles *via* spectrin, and traps them at contact sites between neurons, thus directing newly synthesized proteins to nascent synapses (6). The activation of Src kinase by CD44 stabilizes the structure of the Golgi apparatus by regulating the polymerization of the actin cytoskeleton (7). See the text for further details and references.

Activation of NCAM with soluble NCAM in neurons or NCAM overexpression in heterologous cells induces activation of the mitogen-activated protein kinase (MAPK) pathway (Niethammer et al., 2002; Shin et al., 2002; Figure 1), which triggers phosphorylation and changes activity of multiple substrates in the nucleus (Morrison, 2012). For example, NCAM triggers serine 133 phosphorylation and activation of the transcription factor cyclic AMP response-element binding protein (CREB; Aonurm-Helm et al., 2008; Ditlevsen et al., 2008), which depend in part on the intact MAPK pathway (Schmid et al., 1999). NCAM triggers the MAPK pathway by binding to the fibroblast growth factor receptor (FGFR) and by activating lipid raft-associated kinases, such as Fyn (Niethammer et al., 2002; Bodrikov et al., 2005, 2008). MAPK inhibitors block the NCAM-dependent neurite outgrowth in cultured rat dopaminergic, hippocampal and cerebellar granule neurons, as well as in PC12-E2 cells (Kolkova et al., 2000; Neiiendam et al., 2004). Other IgSF CAMs also trigger the MAPK pathway. MAPK inhibitors inhibit neurite outgrowth induced by soluble fragments of NEGR1 in embryonic mouse cortical neurons (Pischedda and Piccoli, 2015), and block an increase in the expression of MAP2 induced by antibodies against L1 in mouse hippocampal neurons (Poplawski et al., 2012). The MAPK pathway is also triggered by other CAM families, such as cadherins (Yasuda et al., 2007; Lelievre et al., 2012) and integrins (Dalton et al., 2020). Interestingly, the loss of N-cadherin-mediated adhesion also results in the activation of the MAPK pathway in cultured cortical neurons (Ando et al., 2011).

Recent studies indicate that gene expression can also be regulated by the proteolytic fragments of cell surface receptors, which translocate into the nucleus and regulate transcription. For example, the extracellular matrix protein Reelin induces the cleavage of the Reelin receptor ApoER2 by γ-secretase. The intracellular domain of this molecule then translocates to the nucleus, where it regulates transcription by regulating the recruitment of transcription factors to the promoters (Telese et al., 2015). Similarly, intracellular fragments of CAMs have been shown to regulate gene expression (Figure 1). NCAM stimulation results in proteolytic processing of NCAM and formation of a C-terminal fragment of NCAM, consisting of the intracellular domain, transmembrane domain, and stub of the extracellular domain. The NCAM fragments are imported into the nucleus (Kleene et al., 2010; Westphal et al., 2017a), where they regulate gene expression (Westphal et al., 2016, 2017b). Stimulation of L1 with antibodies triggers its cleavage by the protease cathepsin resulting in the generation of a transmembrane fragment, which is then sumoylated and imported to the nucleus (Lutz et al., 2012, 2014). Nuclear levels of neuroglian (Nrg), a *Drosophila* homolog of L1, correlate with the transcript levels of the Myc transcription factor (Kakad et al., 2018). The cleavage of the Down syndrome cell adhesion molecule (DSCAM) and its paralog DSCAM-Like-1 (DSCAML1) by γ-secretase results in the release of their intracellular domains. These domains interact with the importin beta IPO5 *via* a conserved nuclear localization signal. The domains are transported to the nucleus where they regulate genes involved in neurite outgrowth and synapse formation (Sachse et al., 2019).

## CAMs and Regulation of Neuronal Protein Translation

In human endothelial cells, binding of integrins to extracellular matrix-coated beads induces the recruitment of mRNAs and ribosomes to the sites of contacts with the beads (Chicurel et al., 1998) suggesting that CAMs regulate the protein translation machinery. This idea is supported by studies showing that changes in levels of CAMs are accompanied by changes in other proteins. Specifically, levels of the membrane-cytoskeleton linker protein spectrin are reduced in the brains of NCAM deficient mice and increased in cultured hippocampal neurons and heterologous cells overexpressing NCAM (Leshchyns’ka et al., 2003). In cultured embryonic chick sympathetic ganglion cells, inhibition of the NCAM mediated adhesion *via* application of anti-NCAM Fab fragments results in decreased activity of choline acetyltransferase, an enzyme responsible for acetylcholine production (Acheson and Rutishauser, 1988). Levels of the cytoskeletal proteins tubulin and MAP2 are reduced in cultured mouse hippocampal neurons with reduced levels of the neural cell adhesion molecule 2 (NCAM2; Parcerisas et al., 2020). The CAM-dependent changes in protein levels may correlate with changes in transcription. For example, a reduction in MAP2 mRNA levels correlates with reduced MAP2 protein levels in the brains of L1 deficient mice. However, protein levels of both total and phosphorylated ErB2, a tyrosine kinase receptor involved in cell proliferation and migration, are increased, whereas the ErB2 mRNA levels are not altered in the NCAM2 knock-out spinal cord stem cells (Deleyrolle et al., 2015). This data suggests that CAMs also regulate protein levels post-transcriptionally.

The CAM-dependent regulation of translation remains poorly understood. In oligodendrocytes, α6β1-integrins at oligodendrocyte-axon contacts promote translation of the myelin basic protein (MBP) mRNA by releasing the mRNA from the hnRNP-K-containing transport granules (Laursen et al., 2011). Neuroligin 3 controls protein synthesis by regulating the activity of the mammalian target of rapamycin (mTOR) signaling pathway (Figure 1). Deficiency in neuroligin 3 results in hyperactivation of mTOR signaling, increased phosphorylation of ribosomal protein S6, a target of mTOR pathway, and increased rate of translation in cultured rat hippocampal neurons (Xu et al., 2019). Exposure of human high-grade glioma cells to soluble neuroligin 3 secreted by active neurons results in increased phosphorylation of eukaryotic translation initiation factor 4E-binding protein 1 (4E-BP1), which is an mTOR downstream effector (Venkatesh et al., 2015). Expression of adhesion molecule on glia (AMOG) in AMOG deficient human glioma cells results in phosphorylation of Akt with subsequent activation of mTOR signaling (Scheidenhelm et al., 2005). After spinal cord injury, the mTOR activity in neurons is inhibited by homophilic interactions of an IgSF CAM NB3 in corticospinal axons, which binds to NB3 on glial scar-forming cells (Huang et al., 2016). Together, these studies suggest that CAMs regulate protein translation at several levels including the regulation of release of mRNAs from the transport complexes at sites of translation and regulation of the translation machinery.

## CAMs and Regulation of the ER and Golgi Apparatus

Limited evidence suggests that CAMs can also regulate local protein synthesis by regulating the recruitment of organelles required for synthesis, sorting, and delivery of proteins to specific locations within neurons. In developing hippocampal neurons, cell surface NCAM interacts with trans-Golgi network-derived organelles *via* its intracellular domain. NCAM regulates targeting of trans-Golgi network-derived organelles to growth cones and promotes exocytosis and cargo delivery at these sites (Chernyshova et al., 2011). NCAM also captures the trans-Golgi-derived organelles at sites of neurite-to-neurite contacts, which are then transformed into synapses (Sytnyk et al., 2002; Figure 1). PTP1B is an endoplasmic reticulum (ER) anchored tyrosine phosphatase, which interacts with the tyrosine kinase Src at the surface plasma membrane (Monteleone et al., 2012). PTP1B is targeted to the newly forming cell-matrix adhesions (Hernandez et al., 2006) and may link the ER to CAMs at the cell surface, such as NCAM2, which interacts with Src (Sheng et al., 2015). In human fibroblasts, loss of adhesion results in the disorganization of the Golgi apparatus. Re-establishment of adhesion restores the integrity of the Golgi *via* the integrin-dependent activation of Arf1, which recruits the microtubule motor protein dynein to control the Golgi organization (Singh et al., 2018). In hippocampal neurons, a CAM CD44 regulates the positioning of the Golgi in the soma *via* the Src kinase-dependent regulation of the actin cytoskeleton. The knock-down of CD44 causes Golgi fragmentation and dispersion, which is reduced by inhibiting actin polymerization (Skupien et al., 2014; Figure 1).

## Conclusion

Neuronal growth during development and synaptic plasticity in the mature brain depends on the synthesis of new proteins. CAMs are well known as regulators of the neuronal morphology, which mediate the interactions between neurons and the extracellular matrix and neighboring cells. In this review, we draw attention to the growing body of work showing that CAMs also regulate transcription and protein translation and that the protein biosynthesis pathways play a key role in the morphological and functional changes induced by CAMs in neurons. Dysregulation of protein synthesis has been observed in different neurodevelopmental and neurodegenerative disorders, including autism spectrum disorders, fragile X syndrome, and Alzheimer’s disease (Buffington et al., 2014; Ghosh et al., 2020; Lo and Lai, 2020), which are also associated with abnormalities in the expression or processing of CAMs (Leshchyns’ka et al., 2015; Stewart, 2015; Leshchyns’ka and Sytnyk, 2016b; Chmielewska et al., 2019). Understanding the CAM-mediated regulation of protein synthesis can provide further insight into the etiologies of these conditions and, consequently, lead to new therapies.

## Author Contributions

All authors contributed to the literature analysis and writing of the manuscript. All authors contributed to the article and approved the submitted version.

## Conflict of Interest

The authors declare that the research was conducted in the absence of any commercial or financial relationships that could be construed as a potential conflict of interest.
